# Micrometastases in axillary lymph nodes in breast cancer, post-neoadjuvant systemic therapy

**DOI:** 10.1186/s13058-024-01874-x

**Published:** 2024-07-31

**Authors:** Janghee Lee, Seho Park, Soong June Bae, Junghwan Ji, Dooreh Kim, Jee Ye Kim, Hyung Seok Park, Sung Gwe Ahn, Seung Il Kim, Byeong-Woo Park, Joon Jeong

**Affiliations:** 1 Department of Surgery, Dongtan Sacred Heart Hospital, Hallym University, Dongtan, Republic of Korea; 2 Department of Medicine, Yonsei University Graduate School, Seoul, Republic of Korea; 3 Department of Surgery, Severance Hospital, Yonsei University College of Medicine, Seoul, Republic of Korea; 4 Department of Surgery, Gangnam Severance Hospital, Yonsei University College of Medicine, Seoul, Republic of Korea; 5 Institute for Breast Cancer Precision Medicine, Yonsei University College of Medicine, Seoul, Republic of Korea; 6 Department of Surgery, College of Medicine, Seoul St. Marys’s Hospital, The Catholic University of Seoul, Seoul, Republic of Korea

**Keywords:** Breast cancer, Neoadjuvant systemic therapy, Micrometastases, Axillary lymph node, Sentinel lymph node

## Abstract

**Introduction:**

The significance of minimal residual axillary disease, specifically micrometastases, following neoadjuvant systemic therapy (NST) remains largely unexplored. Our study aimed to elucidate the prognostic implications of micrometastases in axillary and sentinel lymph nodes following NST.

**Methods:**

This retrospective study analyzed primary breast cancer patients who underwent surgery after NST from September 2006 through February 2018. All patients received axillary lymph node dissection (ALND), either with or without sentinel lymph node biopsy. Recurrence-free survival (RFS)-associated variables were identified using a multivariate Cox proportional hazard model.

**Results:**

Of the 978 patients examined, 438 (44.8%) exhibited no pathologic lymph node involvement (ypN0) after NST, while 89 (9.1%) had micrometastases (ypN1mi) and 451 (46.7%) had macrometastases (ypN+). Notably, 51.1% of the patients with sentinel lymph node micrometastases (SLNmi) had additional metastases, nearly triple that of SLN-negative patients (*P* < 0.001), and 29.8% of SLNmi patients were upstaged with the ALND. Although ypN1mi was not associated with RFS in patients post-NST (HR, 1.02; 95% CI, 0.42–2.49; *P* = 0.958), SLNmi patients experienced significantly worse RFS compared to SLN-negative patients (hazard ratio [HR], 2.23; 95% confidence intervals [CI], 1.12–4.46; *P* = 0.023). Additional metastases in SLNmi were more prevalent in patients with larger residual breast disease greater than 20 mm, HR-positive/HER2-negative subtype, and low Ki-67 LI (< 14%).

**Conclusions:**

SLNmi is a negative prognostic factor significantly associated with additional non-SLN metastases, while ypN1mi does not influence the prognosis compared to ypN0. Hence, additional ALND may be warranted to confirm axillary nodal status in patients with SLNmi.

**Supplementary Information:**

The online version contains supplementary material available at 10.1186/s13058-024-01874-x.

## Introduction

The prognostic importance of axillary lymph node (LN) metastases in breast cancer has been well established [1]. Traditionally, axillary lymph node dissection (ALND) served as the standard surgical treatment of invasive breast cancer until the 1990s [2]. Since then, sentinel lymph node biopsy (SLNB) has emerged as a viable alternative, offering an accurate prediction of axillary nodal status while mitigating the higher morbidity rates associated with ALND [3, 4].

Nodal status evaluation involves the consideration of metastatic LN size and quantity. The 2002 guidelines introduced micrometastases (0.2 mm < metastatic size ≤ 2.0 mm) as distinct categories [5]. In patients with upfront surgery or in the untreated population, subsequent studies suggested that micrometastases were not correlated with prognosis, and additional ALND did not significantly enhance locoregional recurrence (LRR) and survival rates in patients presenting with sentinel lymph node micrometastases (SLNmi) [6–9].

In the neoadjuvant context, patients with clinically lymph node-positive (cN+) status underwent ALND, independent of their neoadjuvant systemic therapy (NST) response [10]. However, recent trends advocate the judicious avoidance of ALND in patients who transition to clinically lymph node-negative (cN0) status post-systemic therapy, especially in the absence of metastases in a sufficient number (≥ 3) of sentinel lymph node (SLN)s [11–13].

Previous research on minimal residual axillary disease, particularly oncologic outcomes of patients with ypN1mi, after NST has been limited. Furthermore, the evidence regarding additional metastasis in patients with SLNmi is not clear, and consequently, ALND persists as the standard treatment for these patients [14]. This study aims to investigate the significance of pathologic lymph node-micrometastases (ypN1mi) following NST, in comparison to pathologic lymph node-negative (ypN0) or macrometastases (ypN+). We further explore the prognostic implications of SLNmi for the prediction of axillary LN status and survival outcomes.

## Methods

### Study populations

We conducted a retrospective review of primary breast cancer patients from the registries of Gangnam Severance Hospital and Severance Hospital, who underwent surgery following NST between September 2006 and February 2018. The regimen for NST consisted solely of chemotherapy for HER2-negative patients, while both chemotherapy and anti-HER2 therapy were administered to HER2-positive patients. In our cohort, no patients received neoadjuvant endocrine therapy. These patients were clinically diagnosed with stage II or III breast cancer and underwent ALND, with or without SLNB. Exclusion criteria comprised of patients who had upfront surgery, underwent only SLNB, or presented with de novo stage IV disease.

Our study adhered to Good Clinical Practice guidelines and the principles of the Declaration of Helsinki. The institutional review boards (IRB) granted study protocol approval (approval number: 3-2023-0214). The retrospective study design warranted a waiver for the requirement of written informed consent by the IRB.

### Assessment of axillary nodal status

The initial axillary nodal status was evaluated using physical examination, ultrasonography and breast magnetic resonance imaging (MRI). Fine needle aspiration biopsy (FNAB) was conducted on patients where necessary. We classified clinical nodal status based on the clinical staging of the anatomic stage system in the American Joint Commission in Cancer (AJCC) 8th edition, with reference to the results from imaging modalities and FNAB [15]. cN0 was defined as the absence of LN metastases on imaging and physical examination, and cN + was defined as the presence of LN metastases in either imaging or physical examination. Patients with metastatic LN revealed by FNAB were categorized as cN+. Furthermore, we classified suspicious LNs on imaging modalities as cN0 if they yielded negative results on FNAB.

Pathologic nodal status was also evaluated based on the pathologic stage of AJCC 8th edition’s anatomic staging system [15]. We defined metastatic LNs with a size range between > 0.2 mm and ≤ 2 mm as ypN1mi, regardless of the metastatic LN count. LNs exceeding 2 mm were classified as ypN+, and ypN stage was assigned based on the number of LNs, inclusive of micrometastases. Moreover, isolated tumor cells measuring ≤ 0.2 mm were classified as ypN0.

### SLNB and ALND procedures

SLNB was performed using single or dual tracers. For the single tracer technique, Technetium 99, a radioactive substance, was administered periareolarly prior to surgery, and SLNs were identified intraoperatively via a gamma detection system (Neoprobe^®^). The dual tracer method employed both an isosulfan blue dye and Technetium 99 concurrently. The choice of SLNB technique was contingent upon the surgeon’s discretion. SLNs were categorized as one or multiple, and any LN identified by either or both methods was defined as SLN. LNs resected during SLNB without tracer signal were not classified as SLNs.

ALND was characterized by the removal of all LNs in axillary levels I and II. Patients documented to have undergone ALND in surgical records were primarily selected from our registry. Among them, those with fewer than 10 LNs were excluded, based on the assumption that a competent ALND necessitated the removal of 10 or more LNs as defined in previous studies [16–18].

### Statistical analysis

Statistical analyses were conducted using SPSS version 25.0 (IBM Inc., Armonk, NY, USA) and GraphPad Prism, version 9 (GraphPad Software). Differences between groups were assessed using the chi-square test for categorical data and one-way ANOVA for continuous variables, subsequent to confirmation by Levene’s test for equality of variances. The primary outcome was recurrence-free survival (RFS), while overall survival (OS) was analyzed as the secondary outcome. RFS was defined as the interval from breast cancer diagnosis to the initial recurrence, including LRR, distant metastasis, or any cause of death. OS was defined as the duration from breast cancer diagnosis to death from any cause. Kaplan-Meier survival estimations were implemented for RFS and OS, and survival curve group disparities were examined via the log-rank test. Variables associated with RFS and OS were ascertained using a multivariate Cox proportional hazard model, with hazard ratio (HR) and corresponding 95% confidence intervals (CI). The analysis of risk factors for additional metastases in SLNmi patients was performed using a binary logistic regression model. All statistical tests were two-sided, and a *p* value < 0.05 was considered statistically significant.

## Results

**Fig. 1:**
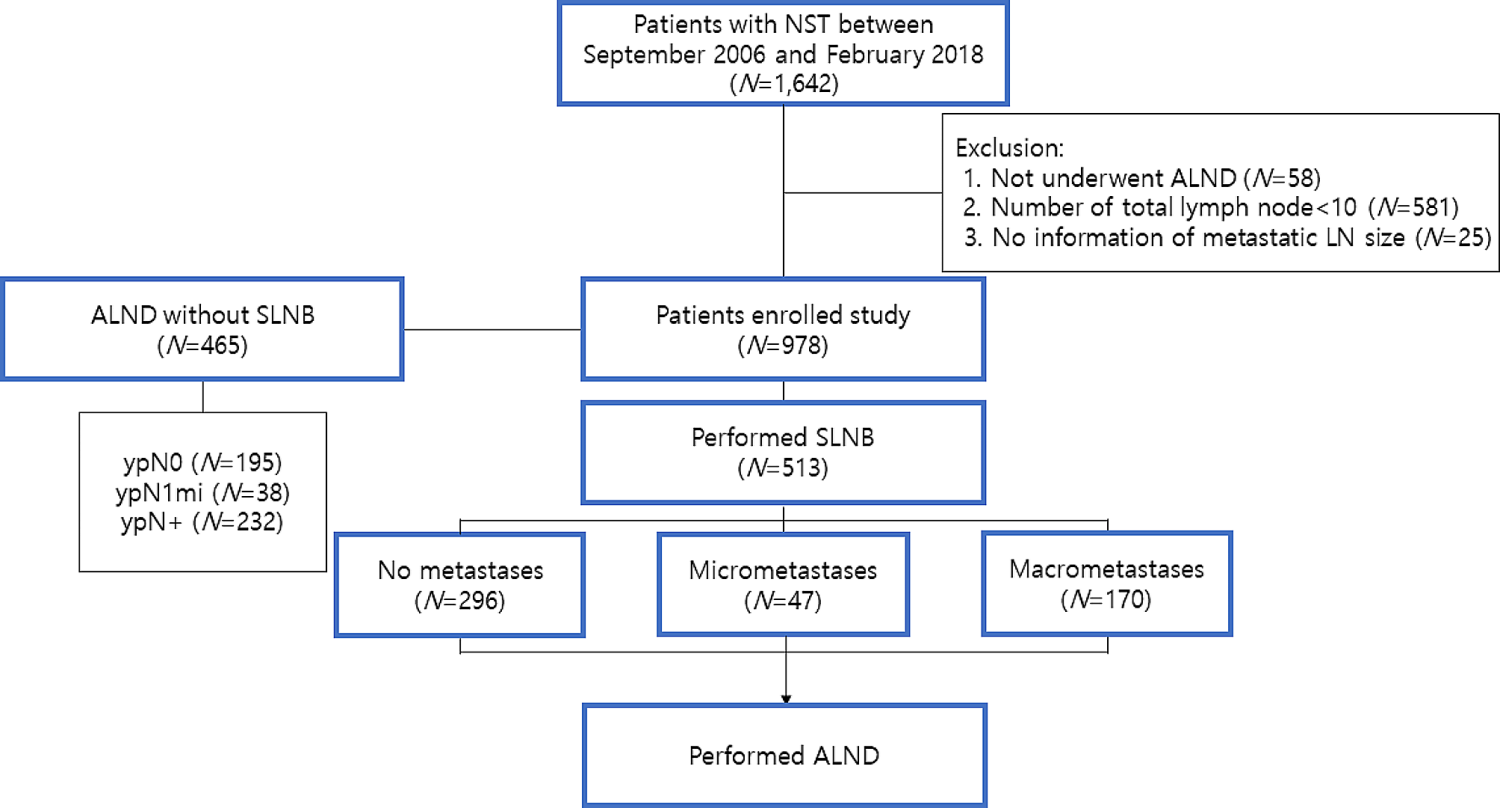
Consort diagram of enrolled patients. NST, neoadjuvant systemic therapy; ALND, axillary lymph node dissection; LN, lymph node; SLNB, sentinel lymph node biopsy

Figure 1 depicts the distribution of axillary surgical procedures among the patients enrolled in our study. Out of the initial 1,642 participants, 664 were excluded due to an inadequate number of axillary LNs or insufficient metastatic LN information. Consequently, 978 patients were analyzed, with a median follow-up duration of 73 months (range, 4-176 months). Among them, 465 (47.5%) patients underwent ALND alone, without SLNB, while 513 (52.5%) patients had SLNB prior to ALND. The clinicopathologic features of the two groups were summarized in eTable 1.

### Baseline characteristics

Of the 978 patients examined, 438 (44.8%) exhibited ypN0 after NST, while 89 (9.1%) had ypN1mi and 451 (46.7%) had ypN+. Table 1 summarizes the clinicopathologic characteristics and differences across these three groups. Among the evaluated cohort, 927 (94.8%) patients were cN + pre-chemotherapy. A significant correlation was observed between pathologic tumor size and axillary nodal status. More than half of the patients with ypN0 achieved a breast pathologic complete response (pCR), while this proportion was significantly lower in the ypN + group (7.3%). The rate of breast pCR in patients with ypN1mi was 29.2%, lower than that in the ypN0 group, but higher than in the ypN + group. Moreover, the quantity of dissected LNs was marginally higher in ypN + patients (*P* = 0.003). The proportion of estrogen receptor (ER)-positive or human epidermal growth factor receptor 2 (HER2)-negative tumors was found to be considerably higher in the ypN1mi and ypN + groups compared to the ypN0 group. The Ki-67 labeling index (LI) was negatively correlated with ypN status. Notably, more patients in the ypN1mi and ypN + groups received adjuvant radiotherapy compared to the ypN0 group.

**Table 1:** Baseline characteristics of all patients

	All Patients (%)	Patients with ypN0 (%)	Patients with ypN1mi (%)	Patients with ypN+ (%)	*P* value
Total	978 (100)	438 (44.8)	89 (9.1)	451 (46.1)	
Age at diagnosis, average (range)	48.5 (20–79)	48.6 (26–79)	47.1 (28–75)	48.8 (20–75)	0.296
Clinical nodal status, initial					0.061
Negative	51 (5.2)	31 (7.3)	3 (3.4)	17 (3.8)	
Positive	927 (94.8)	407 (92.9)	86 (96.6)	434 (96.2)	
Breast surgery					< 0.001
BCS	369 (37.7)	198 (45.2)	40 (44.9)	131 (29.0)	
Mastectomy	609 (62.3)	240 (54.8)	49 (55.1)	320 (71.0)	
Pathologic tumor size (mm)					< 0.001
Breast pCR	283 (28.9)	224 (51.1%)	26 (29.2)	33 (7.3)	
0–20	458 (46.8)	169 (38.6)	49 (55.1)	240 (53.2)	
20–50	194 (19.8)	38 (8.7)	14 (15.7)	142 (31.5)	
>50	43 (4.4)	7 (1.6)	0 (0)	36 (8.0)	
Number of dissected LNs, average (range)	16.7 (10–60)	16.0 (10–38)	16.1 (10–31)	17.4 (10–60)	0.003
ER					< 0.001
Positive	572 (58.5)	186 (42.5)	60 (67.4)	326 (72.3)	
Negative	406 (41.5)	252 (57.5)	29 (32.6)	125 (27.7)	
PR					< 0.001
Positive	431 (44.1)	122 (28.3)	50 (56.2)	259 (57.4)	
Negative	547 (55.9)	316 (72.1)	39 (43.8)	192 (42.6)	
HER2					< 0.001
Negative	638 (65.2)	237 (54.1)	63 (70.8)	338 (74.9)	
Positive	340 (34.8)	201 (45.9)	26 (29.2)	113 (25.1)	
Ki-67 LI, %					< 0.001
<14	370 (37.8)	112 (25.6)	39 (43.8)	219 (48.6)	
≥14	438 (44.8)	224 (51.1)	38 (42.7)	176 (39.0)	
Unknown	170 (17.4)	102 (23.3)	12 (13.5)	56 (12.4)	
Radiotherapy					0.015
Not performed	78 (8.0)	47 (10.7)	4 (4.5)	27 (6.0)	
Performed	900 (92.0)	391 (89.3)	85 (95.5)	424 (94.0)	

BCS, breast-conserving surgery; pCR, pathologic complete response; LN, lymph node; ER, estrogen receptor; PR, progesterone receptor; HER2, human epidermal growth receptor 2; LI, labeling index

### Significance of SLNmi on additional non-SLN metastases

**Fig. 2:**
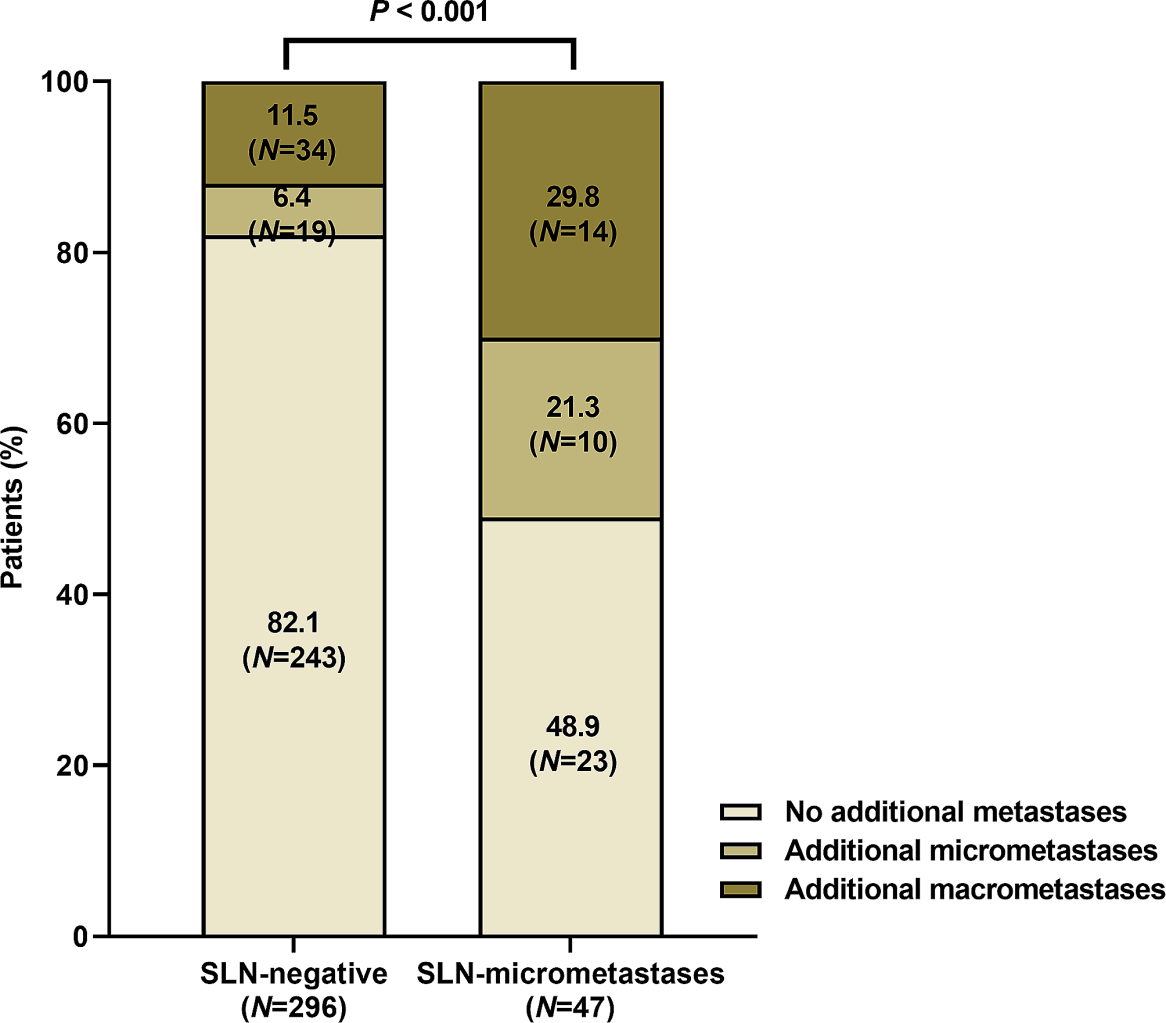
Comparison of additional metastases in non-SLN between SLN-negative and SLN-micrometastases (*P* < 0.001). SLN, sentinel lymph node

Further analyses were conducted on patients who underwent SLNB preceding ALND. Of these patients, 296 (57.7%) were SLN-negative, while 47 (9.2%) exhibited SLNmi (Fig. 2). No significant difference was observed in the average number of removed SLNs between these groups (eTable 2). Patients in the SLNmi category demonstrated larger pathological tumor sizes, higher ER positivity rates, and lower Ki-67 LI compared to SLN-negative patients. Over half of SLNmi patients were identified with additional metastases in non-SLNs, nearly a three-fold increase compared to SLN-negative patients (Fig. 2; *P* < 0.001). Moreover, 29.8% of SLNmi patients were upstaged to ypN + with ALND.

### Survival outcome according to ypN stage

The collective 5-year RFS for all patients was 82%. Broken down by group, the 5year RFS for ypN0, ypN1mi, and ypN + patients were 89%, 87.6%, and 74.1% respectively. Throughout the follow-up period, a total of 183 patients experienced 217 recurrent events. These recurrences manifested as locoregional in 11 patients, systemic in 138 patients, and combined locoregional and systemic in 34 patients. Out of the total patient pool, 85 deaths occurred with a 5-year OS rate of 92.7%. Among the deceased, 70 patients succumbed to breast cancer recurrence, while 15 deaths were attributed to other causes.

The Kaplan-Meier survival curve indicated that patients classified as ypN + demonstrated significantly inferior RFS compared to ypN0 and ypN1mi patients (eFigure 1 A; ypN0 vs. ypN+: *P* < 0.001; ypN1mi vs. ypN+: *P* = 0.008). However, no statistically significant difference was observed in recurrence and survival rates between ypN0 and ypN1mi groups (*P* = 0.455). Furthermore, multivariate Cox regression hazard modeling failed to establish ypN1mi as a significant prognostic factor of RFS in patients undergoing NST (Table 2, HR, 1.70; 95% CI, 0.90–3.22; *P* = 0.104). Factors including macrometastases, large pathological tumor size, high Ki-67 LI (≥ 14%), and lack of radiotherapy were associated with an increased risk of RFS. In terms of OS, the Kaplan-Meier survival curve also indicated a worse prognosis for ypN + patients compared to ypN0 or ypN1mi patients (eFigure 1B; ypN0 vs. ypN+: *P <* 0.001; ypN1mi vs. ypN+: *P* = 0.035). Multivariate analysis continued to highlight macrometastases as a risk factor for OS, whereas micrometastases did not impact survival outcomes (eTable 3; ypN1mi: HR, 1.61; 95% CI, 0.52–4.98; *P* = 0.409; ypN+: HR, 2.88; 95% CI, 1.47–5.66; *P* = 0.002).

**Table 2:** Uni- and multivariate analysis of RFS in patients with NST

	Univariate analysis		Multivariate analysis	
	HR (95% CI)	*P* value	HR (95% CI)	*P* value
Pathologic nodal status				
ypN0	Ref.^**^		Ref.	
ypN1mi	1.26 (0.70–2.26)	0.448	1.70 (0.90–3.22)	0.104
ypN+	2.60 (1.89–3.58)	< 0.001	2.81 (1.85–4.27)	< 0.001
Age at diagnosis^*^	1.00 (0.99–1.02)	0.885		
Breast surgery				
BCS	Ref.		Ref.	
Mastectomy	1.66 (1.21–2.28)	0.002	1.01 (0.70–1.46)	0.964
Pathologic tumor size, mm				
Breast pCR	Ref.		Ref.	
0–20	1.81 (1.17–2.80)	0.007	1.85 (1.05–3.25)	0.033
20–50	3.96 (2.53–6.20)	< 0.001	3.12 (1.71–5.68)	< 0.001
>50	8.31 (4.79–14.43)	< 0.001	5.75 (2.85–11.59)	< 0.001
Number of dissected LNs^*^	1.02 (1.00-1.04)	0.099		
ER				
Positive	Ref.		Ref.	
Negative	1.59 (1.21–2.11)	0.001	1.50 (0.95–2.37)	0.084
PR				
Positive	Ref.		Ref.	
Negative	1.36 (1.02–1.81)	0.035	1.37 (0.86–2.18)	0.191
HER2				
Negative	Ref.			
Positive	0.76 (0.56–1.03)	0.079		
Ki-67 LI, %				
<14	Ref.		Ref.	
≥14	1.78 (1.28–2.47)	0.001	1.83 (1.29–2.61)	0.001
Radiotherapy				
Not performed	Ref.			
Performed	0.62 (0.40–0.95)	0.029	0.55 (0.33–0.91)	0.021

^*^Continuous variable

^**^Reference value

RFS, recurrence free survival; NST, neoadjuvant systemic therapy; HR, hazard ratio; CI, confidence intervals; BCS, breast-conserving surgery; pCR, pathologic complete response; LN, lymph node; ER, estrogen receptor; PR, progesterone receptor; HER2, human epidermal growth receptor 2; LI, labeling index

### Survival outcome of SLNmi patients

**Fig. 3:**
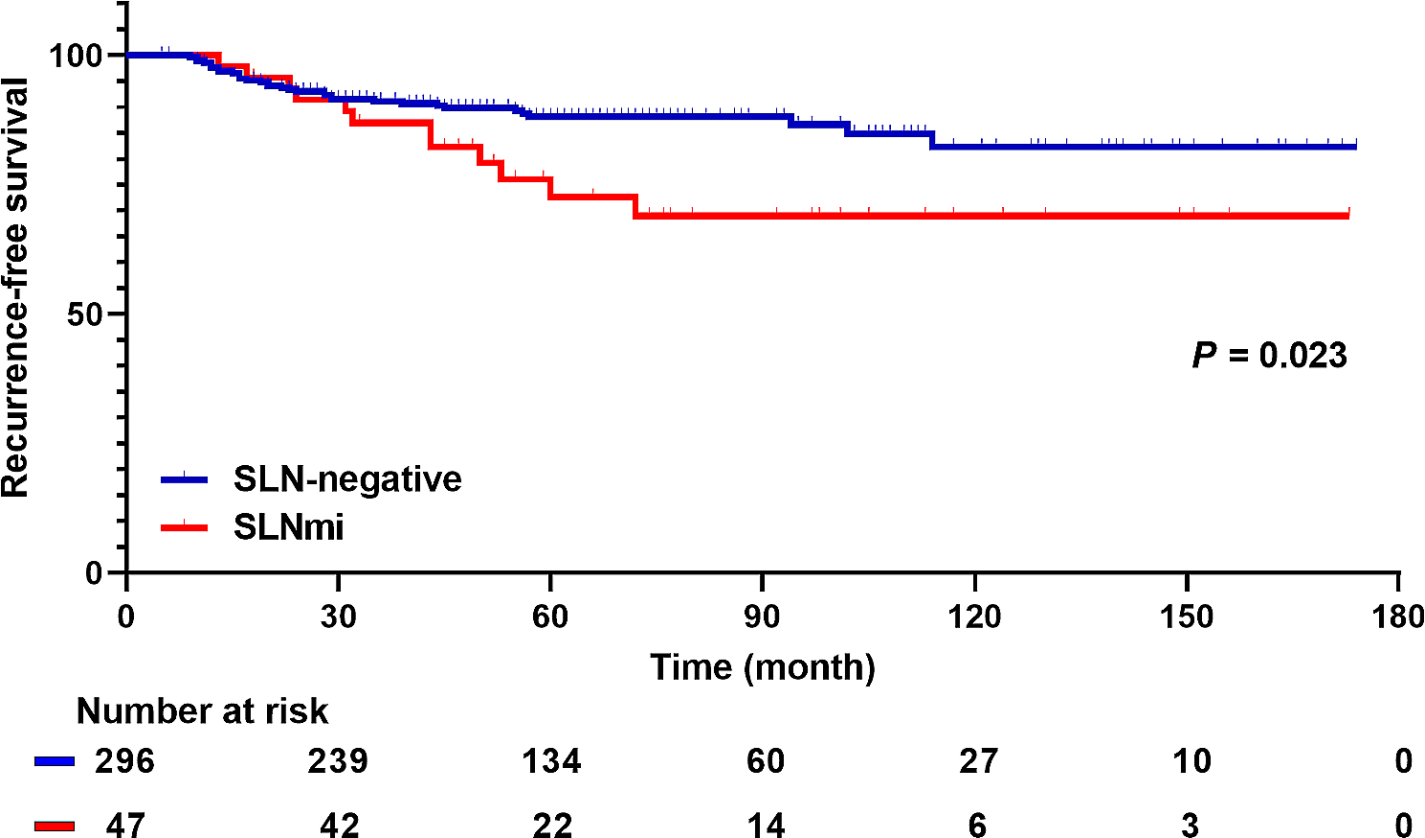
Kaplan-Meier survival curve for RFS of SLN-negative and SLN-micrometastases (*P* = 0.023). RFS, recurrence-free survival; SLN, sentinel lymph node

The 5-year RFS rates for SLN-negative and SLNmi patients were 89.5% and 76.6% respectively. Notably, SLNmi patients had a significantly poorer RFS than SLN-negative patients in the Kaplan-Meier survival analysis (Fig. 3; *P* = 0.023). In the multivariate analysis, SLNmi emerged as a poor prognostic factor for RFS (Table 3; HR, 2.23; 95% CI, 1.12–4.46; *P* = 0.023). Nonetheless, no significant differences were found in OS between SLN-negative and SLNmi patients (eFigure 2 and eTable 4). In addition, we conducted multivariate analysis to identify factors influencing RFS in SLNmi patients (eTable 5). Upstaging to ypN + was not a related factor, and high Ki-67 LI was the only risk factor for recurrence in these patients.

**Table 3:** Uni and multivariate analysis of the effect of SLN-micrometastases on RFS

	Univariate analysis		Multivariate analysis	
	HR (95% CI)	*P* value	HR (95% CI)	*P* value
SLN				
Negative	Ref.^**^		Ref.	
Micrometastases	2.11 (1.09–4.07)	0.026	2.23 (1.12–4.46)	0.023
Age at diagnosis^*^	0.99 (0.96–1.02)	0.466		
Breast surgery				
BCS	Ref.			
Mastectomy	1.13 (0.63–2.01)	0.689		
Pathologic tumor size, mm				
Breast pCR	Ref.		Ref.	
0–20	2.76 (1.23–6.20)	0.014	3.33 (1.31–8.42)	0.011
20–50	5.39 (2.26–12.86)	< 0.001	5.63 (2.11–15.03)	0.001
>50	38.57 (7.97-186.66)	< 0.001	39.48 (7.54-206.74)	< 0.001
ER				
Positive	Ref.		Ref.	
Negative	1.81 (1.01–3.25)	0.045	1.55 (0.79–3.05)	0.203
PR				
Positive	Ref.			
Negative	1.43 (0.77–2.64)	0.260		
HER2				
Negative	Ref.			
Positive	0.77 (0.41–1.47)	0.431		
Ki-67 LI, %				
<14	Ref.		Ref.	
≥14	2.40 (1.18–4.91)	0.016	2.58 (1.17–5.68)	0.019
Radiotherapy				
No	Ref.			
Yes	1.73 (0.53–5.60)	0.363		

^*^Continuous variable

^**^Reference value

SLN, sentinel lymph node; RFS, recurrence free survival; HR, hazard ratio; CI, confidence intervals; BCS, breast-conserving surgery; pCR, pathologic complete response; ER, estrogen receptor; PR, progesterone receptor; HER2, human epidermal growth receptor 2; LI, labeling index

### Risk factors associated with additional metastases in SLNmi patients

Risk factors associated with additional metastases in SLNmi patients were investigated. Additional metastases were more prevalent in patients with pathological tumor size > 20 mm, ER-positive/HER2-negative subtype, and a low Ki-67 LI (< 14%) (eTable 6). Notably, only 30.0% (3/10) patients with HER2-overexpressing breast cancer and 18.2% (2/11) of patients with triple-negative breast cancer (TNBC) had additional metastases. Whereas 73.1% (19/26) of ER-positive/HER2-negative patients demonstrated additional metastases (eTable 7). In the presence of SLNmi, ER-positive/HER2-negative subtype had a significantly higher rate of additional metastases compared to other subtypes (*P* = 0.003). Conversely, no significant difference was observed in the incidence of additional metastases among patients with SLN-macrometastases, irrespective of subtype (*P* = 0.079).

## Discussion

Our study showed that patients with ypN0 and ypN1mi had comparable RFS and OS outcomes, while those with ypN + had a negative impact on oncologic outcomes. Notably, axillary LN status could not be accurately ascertained solely by the micrometastases of SLN. Instances of SLNmi often coincided with additional LN metastases and correlated with worse RFS compared to patients who were SLN-negative. Consequently, our findings suggest that axillary staging via ALND in patients with SLNmi should be considered following NST.

The association between residual axillary disease and prognosis is well-documented [19–21]. We hypothesized the existence of a subgroup of patients who might exhibit a more favorable prognosis, even in the presence of residual axillary tumor burden, such as micrometastases. Our findings substantiate this, demonstrating that patients with ypN1mi have a more favorable prognosis compared to those with ypN+, and their oncologic outcomes are on par with those of ypN0. Prior to our investigation, only two retrospective studies, to the best of our knowledge, examined residual nodal burden. Nijinatten et al. studied prognosis as a function of metastatic LN size in a single institution [22], in which all patients were cN + and 3.8% of patients were classified as ypNitc/mi. They reported a similar prognosis for ypNitc/mi and ypN0 patients, superior to that of ypN + patients. Our results align with these findings within a similar patient population, despite a higher ypN1mi percentage (9.1%) in our study. Conversely, Wong et al. contended that post-NST patients with isolated tumor cells or micrometastases had a worse prognosis than those with ypN0 in both a single institution cohort and the National Cancer Database (NCDB) [23]. They reported ypN1mi rates of 9.1% and 4.7% in their respective cohorts. However, their study included patients who underwent SLNB without ALND in survival analysis. In contrast, our study exclusively enrolled patients who had 10 or more axillary LNs dissected to optimally evaluate axillary nodal status.

SLNB is a valuable intraoperative tool for predicting axillary LN metastases, though it is observed to yield higher rates of false negatives in NST patients compared to those in a primary surgical setting [24, 25]. The SENTinel NeoAdjuvant (SENTINA) trial demonstrated a FNR of 18.5% when two SLNs were removed, despite clinical conversion to node negativity post-NST [11]. In our cohort, the mean number of removed SLNs was 2.62, resulting in an elevated FNR of 23.8%. Furthermore, we noted that 17.9% of SLN-negative patients had verifiable axillary LN metastases.

Our findings indicate a substantially higher incidence of additional metastases in patients with SLNmi relative to those with SLN-negative results. Prior to our investigation, Moo et al. documented that 64% of SLNmi patients exhibited non-SLN metastases, a significantly higher figure than the 17% observed in SLN-negative patients [26]. Weiss et al. also reported that 29.4% of cN1 patients with SLNmi had non-SLN metastases, which was not statistically different from patients with SLN-macrometastases [27]. Our study corroborated these findings, revealing additional metastases in 51.1% of SLNmi patients, 58.3% of which were macrometastases. However, in the adjuvant setting, specifically within the American College of Surgeons Oncology Group Z0011 trial, the incidence of additional metastases among SLNmi was considerably lower, at roughly 10% [28]. This discrepancy suggests a heightened likelihood of additional metastases in NST patients with SLNmi compared to those in the adjuvant setting. In a novel discovery, we also found that SLNmi patients exhibited lower RFS rates than SLN-negative patients. Collectively, these findings suggest the necessity of ALND in patients with SLNmi following NST.

In the analysis of risk factors linked to additional metastases in SLNmi patients, a correlation was observed with pathological tumor size exceeding ypT2, ER-positive/HER2-negative subtype, and low Ki-67 LI (≤ 14%). These findings can be contextualized within the sphere of NST responsiveness. It is well established that ER-positive/HER2-negative tumors present a lower overall response rate [29, 30]. Additionally, low levels of Ki-67 LI associated with reduced pCR rates [31, 32]. Consequently, it can be inferred that SLNB accuracy is interrelated with NST response, and SLNmi is more likely to exhibit additional metastases in patients demonstrating a suboptimal response to chemotherapy. In the study by Weiss et al., it was also reported that all SLNmi patients with additional metastases had hormone receptor-positive subtype [27].

One limitation of this study includes the potential bias stemming from its retrospective design and small sample size of SLNmi patients. However, we were able to source relatively uniform and reliable clinicopathological and prognosis data from two institutions and endeavored to accurately evaluate the axillary nodal status. Nonetheless, we were unable to consider the clinical nodal status after NST as determined by imaging modalities. Future studies combining SLNB results with chemotherapy response evaluations via imaging examination in SLNmi cases may be necessary, as some studies suggest that the accuracy of axillary nodal status prediction can be enhanced when imaging examination results are integrated with SLNB outcomes [33]. Another limitation is the inclusion of cN0 patients in our study cohort, which introduces the possibility of bias in the analysis. However, cN0 patients constitute only 5.2% of the entire cohorts, and since there was no statistical difference between the ypN stage groups, we think their impact is likely to be minimal. enrolled patients. In addition, due to the retrospective nature, the authors did not directly participate in the confirmation of micrometastases detection in final pathology. However, the two institutions where the patients were enrolled have a common protocol for detection of LN metastases, and this has been reconfirmed through consultation with experienced breast pathologist.

In conclusion, SLNmi is an adverse prognostic indicator as a critical predictor of additional metastases, while ypN1mi does not significantly impact patient prognosis when compared with ypN0. Especially, in patients with a poor NST response, SLNmi increase the possibility of additional metastases. As such, additional ALND should be considered for verifying axillary nodal status in patients presenting with SLNmi.

### Electronic supplementary material

Below is the link to the electronic supplementary material.

Supplementary Material 1

## Data Availability

No datasets were generated or analysed during the current study.

## Abbreviations

LN: Lymph node
ALND: Axillary lymph node dissection
SLNB: Sentinel lymph node biopsy
LRR: Locoregional recurrence
SLNmi: Sentinel lymph node micrometastases
cN: clinically lymph node
NST: Neoadjuvant systemic therapy
cN0: clinically lymph node-negative
SLN: Sentinel lymph node
ypN1mi: pathologic lymph node-micrometastases
ypN0: pathologic lymph node-negative
ypN+: pathologic lymph node-macrometastases
IRB: Institutional review boards
MRI: Magnetic resonance imaging
FNAB: Fine needle aspiration biopsy
AJCC: American Joint Commission in Cancer
RFS: Recurrence-free survival
OS: Overall survival
HR: Hazard ratio
CI: Confidence intervals
pCR: pathologic complete response
ER: Estrogen receptor
HER2: Human epidermal growth factor receptor 2
LI: Labeling index
TNBC: Triple negative breast cancer
NCDB: National Cancer Database
SENTINA: SENTinel NeoAdjuvant
BCS: Breast-conserving surgery
PR: Progesterone receptor
