# Supplementary material for: Micrometastases in axillary lymph nodes in breast cancer, post-neoadjuvant systemic therapy
Source: Breast Cancer Res. 2024 Jul 31;26:120. doi: 10.1186/s13058-024-01874-x (PMC11293213; doi:10.1186/s13058-024-01874-x)
Supplement: Supplementary file 1 — Supplementary Material 1 [file 13058_2024_1874_MOESM1_ESM.docx]

**eTable 1.** Comparison of clinicopathologic features between patients who underwent ALND alone and those who underwent ALND after SLNB

|  | Patients without SLNB (%) | Patients with SLNB (%) | *P* value |
| --- | --- | --- | --- |
| Total | 465 (47.5%) | 513 (52.5%) |  |
| Age at diagnosis, average (range) | 49.3 (20-79) | 47.9 (27-75) | 0.021 |
| Clinical nodal status, initial |  |  | 0.037 |
| Negative | 17 (3.7) | 34 (6.6) |  |
| Positive | 448 (96.3) | 479 (93.4) |  |
| Breast surgery |  |  | < 0.001 |
| BCS | 149 (32.0) | 220 (42.9) |  |
| Mastectomy | 316 (68.0) | 293 (57.1) |  |
| Pathologic tumor size (mm) |  |  | 0.039 |
| Breast pCR | 129 (27.7) | 154 (30.0) |  |
| 0-20 | 207 (44.5) | 251 (48.9) |  |
| 20-50 | 101 (21.7) | 93 (18.1) |  |
| >50 | 28 (6.0) | 15 (2.9) |  |
| Number of dissected LNs, average (range) | 17.1 (10-60) | 16.3 (10-38) | 0.059 |
| ER |  |  | 0.001 |
| Positive | 246 (52.9) | 326 (63.5) |  |
| Negative | 219 (47.1) | 187 (36.5) |  |
| PR |  |  | 0.096 |
| Positive | 192 (41.3) | 239 (46.6) |  |
| Negative | 273 (58.7) | 274 (53.4) |  |
| HER2 |  |  | < 0.001 |
| Negative | 272 (58.5) | 366 (71.3) |  |
| Positive | 193 (41.5) | 147 (28.7) |  |
| Ki-67 LI, % |  |  | 0.009 |
| <14 | 152 (32.7) | 218 (42.5) |  |
| ≥14 | 220 (47.3) | 218 (42.5) |  |
| Unknown | 93 (20.0) | 77 (15.0) |  |
| Radiotherapy |  |  | 0.334 |
| Not performed | 33 (7.1) | 45 (8.8) |  |
| Performed | 432 (92.9) | 468 (91.2) |  |

ALND, axillary lymph node dissection; SLNB, sentinel lymph node biopsy; BCS, breast-conserving surgery; pCR, pathologic complete response; LN, lymph node; ER, estrogen receptor; PR, progesterone receptor; HER2, human epidermal growth receptor 2; LI, labeling index

**eTable 2.** Comparison of clinicopathologic characteristics between SLN-negative and SLNmi

|  | SLN-negative (%) | SLNmi (%) | *P* value |
| --- | --- | --- | --- |
| Age at diagnosis, average (range), yrs | 48.1 (27-75) | 45.7 (28-69) | 0.113 |
| Number of SLNs, average (range) | 2.52 (1-12) | 2.47 (1-8) | 0.851 |
| Number of dissected LNs, average (range) | 15.82 (10-38) | 15.64 (10-26) | 0.821 |
| Breast surgery |  |  | 0.065 |
| BCS | 150 (50.7) | 17 (36.2) |  |
| Mastectomy | 146 (49.3) | 30 (63.8) |  |
| Pathologic tumor size, mm |  |  | 0.015 |
| Breast pCR | 132 (44.6) | 10 (21.3) |  |
| 0-20 | 124 (41.9) | 26 (55.3) |  |
| 20-50 | 38 (12.8) | 11 (23.4) |  |
| >50 | 2 (0.7) | 0 (0) |  |
| ER |  |  | 0.034 |
| Positive | 159 (53.7) | 33 (70.2) |  |
| Negative | 137 (46.3) | 14 (29.8) |  |
| PR |  |  | 0.002 |
| Positive | 107 (36.1) | 28 (59.6) |  |
| Negative | 189 (63.9) | 19 (40.4) |  |
| HER2 |  |  | 0.067 |
| Negative | 193 (65.2) | 37 (78.7) |  |
| Positive | 103 (34.8) | 10 (21.3) |  |
| Ki-67 LI, % |  |  | 0.041 |
| < 14 | 97 (32.8) | 25 (53.2) |  |
| ≥ 14 | 144 (48.6) | 19 (40.4) |  |
| Unknown | 55 (18.6) | 3 (6.4) |  |
| Radiotherapy |  |  | 0.179 |
| Not performed | 31 (10.5) | 2 (4.3) |  |
| Performed | 265 (89.5) | 45 (95.7) |  |

SLN sentinel lymph node; LN, lymph node; BCS, breast-conserving surgery; pCR, pathologic complete response; ER, estrogen receptor; PR, progesterone receptor; HER2, human epidermal growth receptor 2; LI, labeling index

**eTable 3.** Uni- and multivariable analysis of OS in patients with NST

|  | Univariate analysis | | Multivariable analysis | |
| --- | --- | --- | --- | --- |
|  | HR (95% CI) | *P* value | HR (95% CI) | *P* value |
| Pathologic nodal status |  |  |  |  |
| ypN0 | Ref.^**^ |  | Ref. |  |
| ypN1mi | 1.10 (0.42-2.92) | 0.847 | 1.61 (0.52-4.98) | 0.409 |
| ypN+ | 2.79 (1.69-4.59) | < 0.001 | 2.88 (1.47-5.66) | 0.002 |
| Age at diagnosis^*^ | 1.02 (1.00-1.04) | 0.137 |  |  |
| Breast surgery |  |  |  |  |
| BCS | Ref. |  | Ref. |  |
| Mastectomy | 2.72 (1.56-4.77) | < 0.001 | 1.57 (0.80-3.10) | 0.194 |
| Pathologic tumor size, mm |  |  |  |  |
| Breast pCR | Ref. |  | Ref. |  |
| 0-20 | 2.84 (1.26-6.43) | 0.012 | 4.41 (1.28-15.20) | 0.019 |
| 20-50 | 6.11 (2.67-13.98) | < 0.001 | 6.71 (1.87-24.04) | 0.003 |
| >50 | 19.96 (8.20-48.57) | < 0.001 | 13.67 (3.50-53.39) | < 0.001 |
| Number of dissected LN^*^ | 1.05 (1.02-1.08) | 0.002 | 1.01 (0.98-1.05) | 0.513 |
| ER |  |  |  |  |
| Positive | Ref. |  | Ref. |  |
| Negative | 3.03 (1.93-4.77) | < 0.001 | 2.08 (0.97-4.46) | 0.060 |
| PR |  |  |  |  |
| Positive | Ref. |  | Ref. |  |
| Negative | 0.45 (0.28-0.72) | 0.001 | 2.22 (0.92-5.40) | 0.078 |
| HER2 |  |  |  |  |
| Negative | Ref. |  |  |  |
| Positive | 0.81 (0.51-1.29) | 0.373 |  |  |
| Ki-67 LI, % |  |  |  |  |
| ≤14 | Ref. |  | Ref. |  |
| >14 | 3.81 (1.98-7.33) | < 0.001 | 3.02 (1.50-6.08) | 0.002 |
| Radiotherapy |  |  |  |  |
| Not performed | Ref. |  | Ref. |  |
| Performed | 0.49 (0.27-0.91) | 0.024 | 0.58 (0.27-1.26) | 0.168 |

^*^Continuous variable

^**^Reference value

OS, overall survival; NST, neoadjuvant systemic therapy; HR, hazard ratio; CI, confidence intervals; BCS, breast-conserving surgery; LN, lymph node; pCR, pathologic complete response; ER, estrogen receptor; PR, progesterone receptor; HER2, human epidermal growth receptor 2; LI, labeling index

**eTable 4.** Cox regression analysis of SLN-micrometastases for OS

|  | Univariable analysis | | Multivariable analysis | |
| --- | --- | --- | --- | --- |
|  | HR (95% CI) | *P* value | HR (95% CI) | *P* value |
| SLN |  |  |  |  |
| Negative | Ref.^**^ |  | Ref. |  |
| Micrometastases | 1.84 (0.59-5.70) | 0.293 | 2.82 (0.82-9.72) | 0.100 |
| Age at diagnosis^*^ | 1.01 (0.96-1.06) | 0.768 |  |  |
| Breast surgery |  |  |  |  |
| BCS | Ref. |  |  |  |
| Mastectomy | 1.61 (0.58-4.43) | 0.358 |  |  |
| Pathologic tumor size, mm |  |  |  |  |
| Breast pCR | Ref. |  | Ref. |  |
| ≤ 20 | 4.10 (0.87-19.33) | 0.074 | 8.38 (1.01-69.48) | 0.049 |
| 20-50 | 5.94 (1.09-32.45) | 0.040 | 8.65 (0.89-84.04) | 0.063 |
| > 50 | 187.03 (23.76-1472.30) | < 0.001 | 234.43 (18.80-2923.76) | < 0.001 |
| ER |  |  |  |  |
| Positive | Ref. |  | Ref. |  |
| Negative | 3.03 (1.05-8.72) | 0.040 | 2.55 (0.74-8.79) | 0.139 |
| PR |  |  |  |  |
| Positive | Ref. |  |  |  |
| Negative | 1.48 (0.51-4.26) | 0.467 |  |  |
| HER2 |  |  |  |  |
| Negative | Ref. |  |  |  |
| Positive | 0.65 (0.21-2.02) | 0.457 |  |  |
| Ki-67, % |  |  |  |  |
| < 14 | Ref. |  | Ref. |  |
| ≥ 14 | 8.42 (1.09-64.76) | 0.041 | 6.94 (0.81-59.28) | 0.077 |
| Radiotherapy |  |  |  |  |
| Not performed | Ref. |  |  |  |
| Performed | 1.72 (0.23-13.06) | 0.599 |  |  |

^*^Continuous variable

^**^Reference value

SLN, sentinel lymph node; OS, overall survival; HR, hazard ratio; CI, confidence intervals; BCS, breast-conserving surgery; pCR, pathologic complete response; ER, estrogen receptor; PR, progesterone receptor; HER2, human epidermal growth receptor 2; LI, labeling index

**eTable 5.** Multivariate analysis of factors associated with RFS in SLNmi patients

|  | HR (95% CI) | *P* value |
| --- | --- | --- |
| Pathologic nodal status |  |  |
| ypN1mi | Ref.^**^ |  |
| ypN+ | 2.53 (0.38-17.06) | 0.340 |
| Age at diagnosis^*^ | 1.04 (0.98-1.10) | 0.229 |
| Breast surgery |  |  |
| Breast conserving surgery | Ref. |  |
| Total mastectomy | 0.60 (0.08-4.57) | 0.621 |
| Pathologic tumor size |  |  |
| Breast pCR | Ref. |  |
| ≤ 20 | 7.26 (0.61-86.16) | 0.116 |
| 20-50 | 6.57 (0.35-122.23) | 0.207 |
| ER |  |  |
| Positive | Ref. |  |
| Negative | 0.30 (0.03-3.39) | 0.327 |
| PR |  |  |
| Positive | Ref. |  |
| Negative | 6.30 (0.62-63.89) | 0.119 |
| HER2 |  |  |
| Negative or Unknown | Ref. |  |
| Positive | 1.29 (0.26-6.33) | 0.752 |
| Ki-67 LI, % |  |  |
| < 14 | Ref. |  |
| ≥ 14 | 7.22 (1.20-43.39) | 0.031 |
| Radiotherapy |  |  |
| Not performed | Ref. |  |
| Performed | NE | 0.989 |

^*^Continuous variable

^**^Reference value

RFS; recurrence-free survival; SLN, sentinel lymph node; HR, hazard ratio; CI, confidence intervals; pCR, pathologic complete response; ER, estrogen receptor; HER2, human epidermal growth factor receptor 2; TNBC, triple negative breast cancer; LI, labeling index

**eTable 6.** Risk factors associated with additional metastases in SLN-micrometastases patients

|  | OR (95% CI) | *P* value |
| --- | --- | --- |
| Age at diagnosis^*^ | 1.00 (0.94-1.05) | 0.904 |
| Clinical nodal status |  |  |
| Negative | Ref.^**^ |  |
| Positive | 0.32 (0.03-3.31) | 0.338 |
| Pathologic tumor size, mm |  |  |
| Breast pCR | Ref. |  |
| 0-20 | 4.67 (0.83-26.34) | 0.081 |
| 20-50 | 10.67 (1.39-82.03) | 0.023 |
| Subtype |  |  |
| ER(+)/HER2(-) | Ref. |  |
| HER2(+) | 0.16 (0.03-0.79) | 0.024 |
| TNBC | 0.08 (0.01-0.48) | 0.005 |
| Ki-67 LI, % |  |  |
| <14 | Ref. |  |
| ≥14 | 0.22 (0.06-0.78) | 0.020 |

^*^Continuous variable

^**^Reference value

SLN, sentinel lymph node; OR, odd ratio; CI, confidence intervals; pCR, pathologic complete response; ER, estrogen receptor; HER2, human epidermal growth factor receptor 2; TNBC, triple negative breast cancer; LI, labeling index

**eTable 7.** Additional metastases according to subtype

|  | Additional metastases (*N*/Total, %) | | | |
| --- | --- | --- | --- | --- |
|  | ER+/HER2- | HER2+ | TNBC | *P* value |
| SLN-negative | 34/96 (35.4) | 12/103 (11.7) | 7/97 (7.2) | < 0.001 |
| SLNmi | 19/26 (73.1) | 3/10 (30.0) | 2/11 (18.2) | 0.003 |
| SLN-macrometastases | 58/110 (52.7) | 20/34 (58.8) | 20/26 (76.9) | 0.079 |

SLN, sentinel lymph node; ER, estrogen receptor; PR, progesterone receptor; HER2, human epidermal growth receptor 2


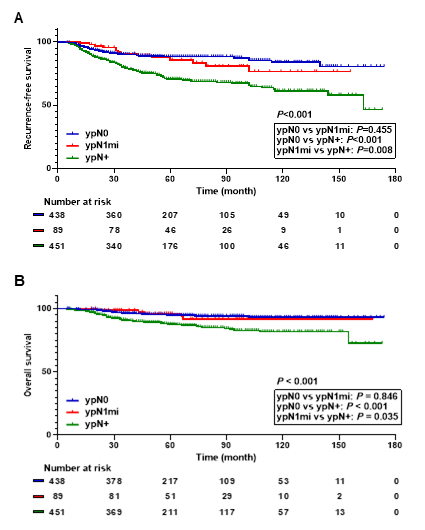


**eFigure 1.** Kaplan-Meier survival curve analysis of survival outcomes based on pathologic nodal status. (A) Recurrence-free survival (*P* < 0.001); (B) Overall survival (*P* < 0.001)


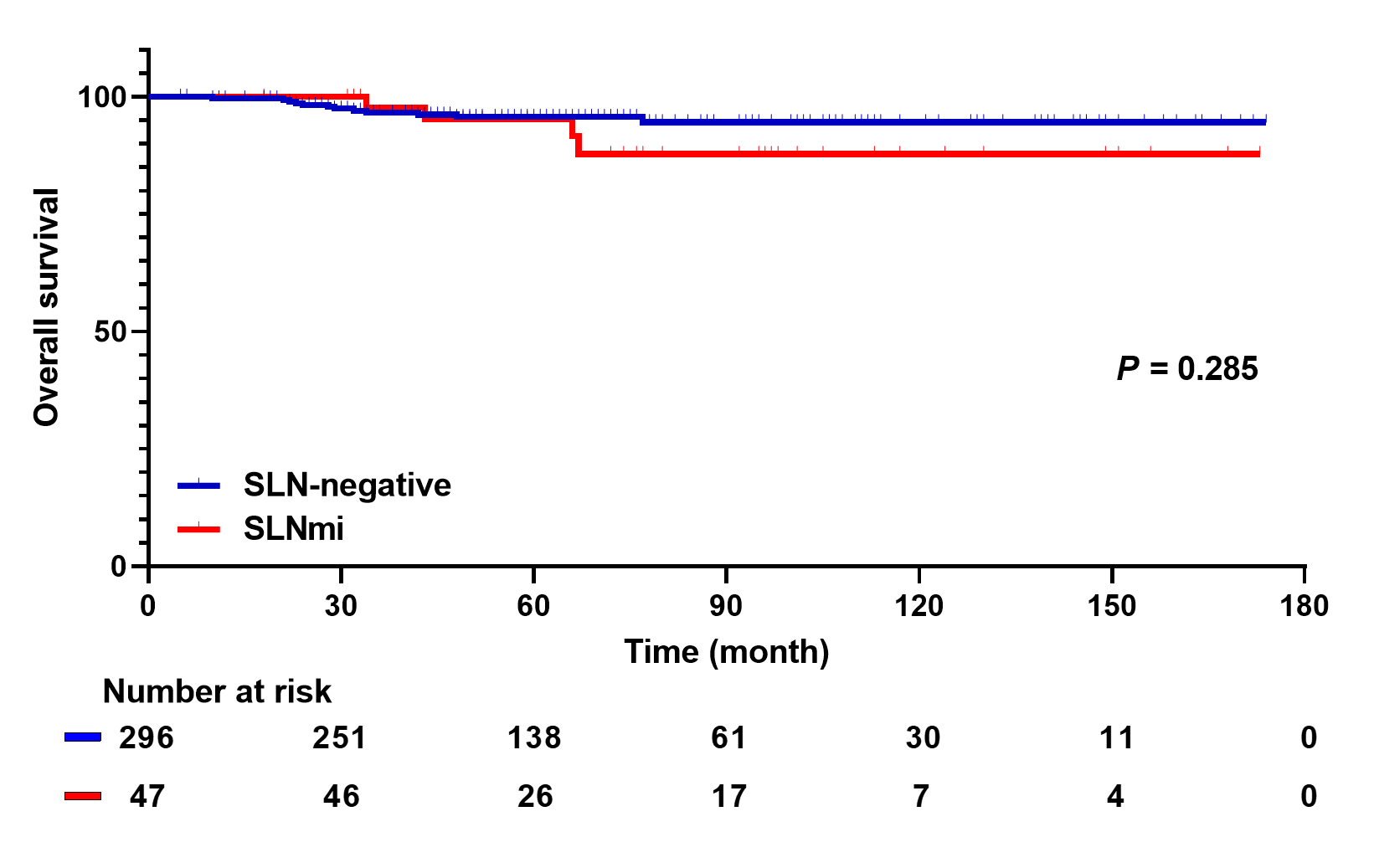


**eFigure 2.** Kaplan-Meier survival curve for OS of SLN-negative and SLN-micrometastases (*P* = 0.285)

OS, overall survival; SLN, sentinel lymph node
